# The impact of women’s individualization on commercial insurance purchase behavior: evidence from China

**DOI:** 10.3389/fpubh.2025.1627096

**Published:** 2025-10-07

**Authors:** Li Wang, Lifei Gao, Shuixing Liu, Wenzhong Li, Yunpeng Bai

**Affiliations:** ^1^School of Finance, Shanxi University of Finance and Economics, Taiyuan, China; ^2^School of Economics, Beijing Technology and Business University, Beijing, China; ^3^School of Urban Economics and Public Administration, Capital University of Economics and Business, Beijing, China; ^4^School of Finance, Capital University of Economics and Business, Beijing, China; ^5^Bank of China Limited Hebei Branch, Shijiazhuang, China

**Keywords:** women’s individualization, risk, health, income, cognition, commercial insurance

## Abstract

**Introduction:**

Women’s individualization, which enables them to become unshackled from their family bondage and become more independent, amplifies exposure to occupational hazards, gender discrimination, work–family conflicts, and health risks, increasing the need for risk mitigation. Providing pioneering empirical evidence, this study quantifies how individualization reshapes Chinese women’s commercial insurance demand by integrating social theory with risk management principles.

**Methods:**

Based on the latest three-wave data from the China Comprehensive Social Survey, we analyze the impact of women’s individualization on commercial insurance purchasing behavior using various methods such as the probit model, instrumental variable method, and propensity score matching.

**Results:**

Women’s individualization can significantly increase the probability of purchasing commercial medical insurance by 1.430% and commercial endowment insurance by 0.743%. This effect operates through heightened risk management awareness, increased purchasing power, and enhanced cognitive ability. The impact is stronger among employed women, those with higher wealth, poorer health, younger and middle-aged women, and women in central and eastern China.

**Conclusion:**

This study reveals that women’s individualization motivates them to transfer personal risks through insurance purchasing, demonstrating how social transformation shapes financial decision-making. Crucially, findings inform stakeholders: policymakers are advised to prioritize insurance subsidies for vulnerable women and adapt regulations to regional market needs. Insurers should innovate tailored products addressing individualized women’s distinct risk profiles. Women should proactively mitigate personal risks through tailored insurance solutions. Researchers are offered a novel framework linking social transformation to financial behavior, urging further cross-cultural investigation.

## 1 Introduction

Ulrich Beck’s individualization theory posits that reflexive modernization liberates individuals from traditional social structures such as family, class, gender roles, while simultaneously increasing dependence on institutional systems (1, 2). This dynamic, termed “institutionalized individualism,” transfers risk responsibilities to individuals (1). Female individualization, which refers to the liberation of women, is a typical representative of individualization. For women, the individualization manifests as a shift from “living for others” to “living for themselves” (3). The transition is accompanied by new forms of risk. This study critically examines whether and through what mechanisms female individualization drives women to counteract individualized risks through commercial insurance, a core institutional safeguard within reflexive modernity.

**Figure 1:**
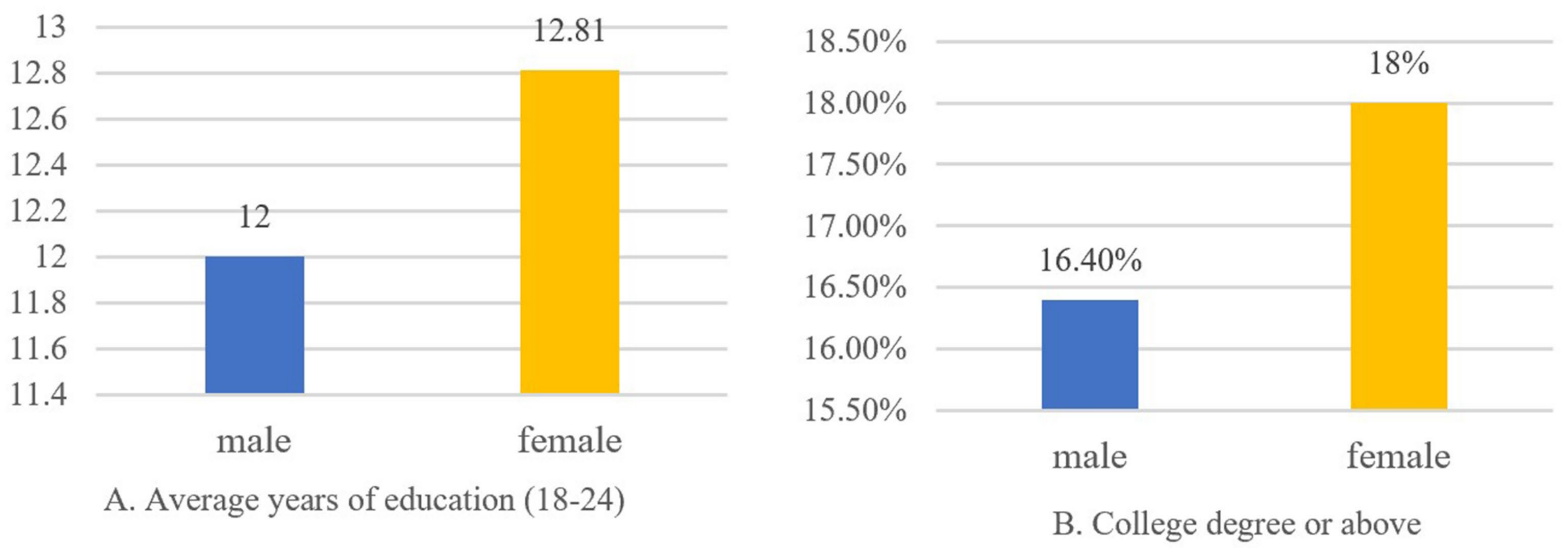
Education level of Chinese women and men in 2020.

**Figure 2:**
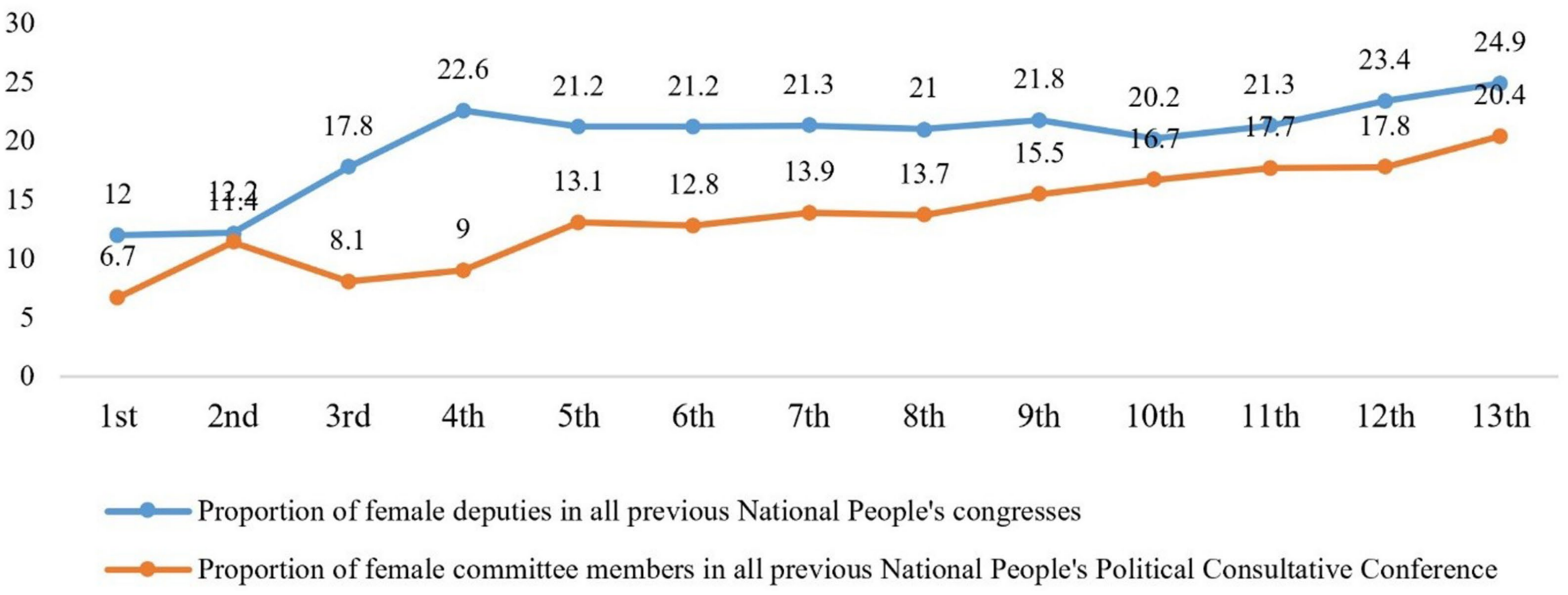
Women’s political status in China.

The rapid transformation of women’s status in China makes it a crucial case for examining both the risks faced by individualized women and how they navigate these challenges. Historically, Chinese women endured systemic gender discrimination under Confucian patriarchy (4). Since 1949, China established legal gender equality, enabling their transition from domestic roles to dual-identity “social persons” in public spheres. This shift significantly reduced dependence on men while dismantling millennium-old gendered divisions of labor (5). In contemporary China, women achieved 68.6% labor force participation in 2021, surpassed men in 18–24 education metrics (Figure 1),^1^ and secured stronger political rights (Figure 2).^2^ Over 80% of major household decisions now involve spousal collaboration, with 94.8% acknowledging women’s equal socioeconomic contributions (6). Yet women’s individualization generates countervailing risks, increasing divorce likelihood (7), unemployment, work–family conflict,^3^ physical and mental health deterioration. Concerningly, 65% report sleep disorders, 58% experience clinical anxiety, and 98.95% of Shanghai white-collar workers showed abnormal health indicators,^4^ while divorce rates persist above 3‰ for five consecutive years.^5^ According to the White Paper on Women’s Health and Risk Management in China (2019), Chinese women demonstrate stronger risk awareness than men, translating to heightened demand for critical illness, medical, accident, and endowment insurance.

Extensive research examines how economic, cultural, and familial structures shape insurance demand. Existing scholarship primarily analyzes risks emerging from female individualization (1) and documents gender-differentiated risk preferences, with women demonstrating heightened risk sensitivity (8, 9) and greater risk aversion (10). Furthermore, empirical work confirms that work–family conflict (11), financial pressures stemming from divorce or widowhood (12), and childcare responsibilities (13) significantly influence women’s insurance behaviors. Chen demonstrated that health insurance has a “marriage lock” function, indicating that commercial insurance can serve as a means of individualized risk management tool for women (14). However, there is insufficient research on how women manage individualization risks. Relevant empirical research is even rarer. We explore the causal relationship between female individualization and commercial insurance purchasing, which belongs to the interdisciplinary research of sociology and economics.

This study investigates whether individualization motivates women to manage risks through commercial insurance adoption. Leveraging nationally representative data from the Chinese General Social Survey (CGSS), we analyze the relationship between female individualization and commercial insurance demand using robust methodologies including probit regression, instrumental variable techniques, propensity score matching, and placebo tests. Results demonstrate that women’s individualization significantly increases their purchase of commercial medical and endowment insurance. Our findings reveal how commercial insurance serves as a critical risk mitigation tool for individualized women, offering practical pathways for navigating modern vulnerabilities.

This research makes three key contributions. First, it establishes an empirical connection between women’s individualization and insurance consumption, providing new evidence on how social changes influence financial behaviors in developing economies. Second, it explores how individualization encourages women to purchase commercial insurance through theoretical analysis and moderating effects. Third, this study offers significant policy implications for mitigating gendered risks within individualized societies.

The remainder of the paper is presented as follows. Section 2 presents the literature review and theoretical analysis. Section 3 presents the data and method. Section 4 explains the results, including the basic regression results, analysis of the endogenous problem, and robustness, mechanism, and heterogeneity analyses. Section 5 makes the discussion. Section 6 presents the conclusion.

## 2 Literature review and theoretical analysis

### 2.1 Theoretical foundation

Individualization theory, originating from Ulrich Beck’s Risk Society (1), argues that modernity erodes traditional social structures, like kinship and class, creating a paradox: liberation from constraints coincides with new reliance on institutional systems like labor markets. This dynamic, termed “institutionalized individualism” (1), transfers risk responsibilities to individuals.

For women, individualization enables transitions from prescribed “living for others” to self-directed “do-it-yourself biographies” (15). On one hand, it concurrently amplifies exposure to labor market precarity and healthcare deficits (15). On the other hand, it erodes traditional safeguards formerly provided through kinship networks. In China’s accelerated modernization context, Yan (16) documents correlated phenomena: rapid female autonomy expansion alongside familial support network fragmentation.

The process of social individualization has escalated individualized risks faced by residents (1). Effective management of these risks first requires their systematic identification – an essential task completed in Section 2.2.1 of this study.

According to foundational risk management theory (17), five core strategies are risk avoidance, risk retention, risk prevention, risk control, risk transfer. Insurance constitutes a critical risk-transfer mechanism for addressing individualized risks (18). By shifting these risks through insurance products, women in risks may better adapt to increasingly individualized societies.

As Beck’s theory says, individualization destroys forced group rules, pushing people to handle risks alone (1). For women, this means facing male-centered risks that used to be handled by families. This necessitates market-based solutions like commercial insurance.

### 2.2 Women’s individualization risk and insurance demands

#### 2.2.1 Women’s individualization risk

Women’s education and employment play an important role in their empowerment and individualization (19). However, individualization can also lead to many risks, such as occupational risk, work–family conflict, and divorce risk, which may threaten women’s physical and mental health.

##### 2.2.1.1 Occupational risk

Long working hours, toxic and harmful chemicals, dust, and noise at work result in obvious health risks to women (20). There is also a risk of job replacement for women, as most are engaged in low-skill jobs that are most likely to be replaced by technology. In addition, women’s unemployment risk is significantly higher due to evident gender discrimination in the labor market (21–23). This forces women to tend to delay marriage and have fewer children to increase their job opportunities (24). As women become increasingly independent and participate in social work, sexual harassment in the workplace occurs, which considerably threatens women’s bodies and minds (25).

##### 2.2.1.2 Work–family conflict

In the process of individualization, women face the dual challenges of working and taking care of their families. The family pattern of modern society has changed from the original “male breadwinner pattern” to “double employment” (26). Women need to balance high-intensity market labor with unpaid housework, such as caring for the old and young, which usually leads to work–family conflict and significantly increases the pressure on women, thus affecting their mental and physical health. Caring for the older adults and children reduces women’s working hours and earnings (27, 28). The increasing work–family conflict has also been reported to hamper women’s willingness to give birth (29).

##### 2.2.1.3 Divorce risk

Women’s individualization increases the risk of divorce. On the one hand, the improvement in women’s independence deepens the degree of deviation from traditional gender identity tropes, and the satisfaction rate of women with their spouse’s financial and domestic contribution decreases (30, 31). On the other hand, individualization increases women’s independence, reduces their dependence on their husbands, and increases their dissatisfaction with their traditional status in marriage, which challenges their husbands’ authority in the family (7, 32).

##### 2.2.1.4 Other risks

The conflict between work and family can suppress women’s fertility (26), thereby increasing women’s endowment risk. The consequence of delaying marriage is that single urban women who are over 30 are subject to a range of punitive actions, including insults, verbal violence, and social exclusion (33). With the higher level of institutional discrimination such as restricted access to justice, unequal rights, household responsibilities, divorce, and inheritance, and women in low- and middle-income countries have relatively high suicide rates (34).

#### 2.2.2 Women’s risk perception and attitudes

Studies have shown that women are more sensitive to risk, and their risk perception is usually much higher than that of men (8, 9). Regarding risk attitudes, biological and socio-cultural factors may lead to women’s conservative attitudes (35). Compared to men, women have also been found to be more risk-averse (10), especially while making financial choices.

#### 2.2.3 Women’s insurance demand

As discussed above, the risks associated with women’s individualization put them under great pressure. Improved risk perception usually urges individuals to take measures to avoid the risk. As high risk causes fear, anxiety, depression, and other negative emotions, people take the initiative to relieve or alleviate this state (36, 37). Affected by the characteristics of high-risk awareness and risk aversion attitudes, women may manage their risks by purchasing commercial insurance.

Previous studies have shown that risk perception can promote insurance demand (38, 39). Work–family conflict increases women’s pressure regarding taking care of older adults, which can encourage them to purchase commercial health insurance (40) and commercial endowment insurance (41). Work–family conflict also reduces women’s willingness to have children, as women have shifted from relying on their children to relying on commercial endowment insurance (11). A reverse relationship exists between the number of children raised by a family and the purchase of commercial endowment insurance (13). In addition, Westland (12) found that women tend to purchase divorce insurance and commercial life insurance to prevent the financial pressure caused by divorce and widowhood.

### 2.3 Women’s individualization and insurance purchasing ability

Individualization can increase women’s income, decision-making power, and cognitive ability, which may improve their ability to purchase insurance. Social labor participation enhances women’s income and objective insurance purchasing power. In addition, with the improvement of women’s status in the family, they have more bargaining power (42) and more decision-making power regarding their healthcare, major household purchases, spending of household earnings, and in other areas (19). Su et al. (43) found that an improvement in women’s bargaining power can significantly improve total household consumption. The increasing income and decision-making power will allow women to purchase insurance freely.

Some scholars have indicated that low awareness and knowledge regarding the insurance product (44) and misperceptions of risk (45) may lead to a low demand for insurance. Similarly, Christelis et al. (46) and Fang et al. (47) suggested that cognitive ability would affect financial asset allocation decisions, including those pertaining to insurance purchases. The more independent the minds of women, the more inclined they are to have a better lifestyle. They break away from family constraints, exercise more, and engage more in social interactions. Some scholars have found that physical exercise (48, 49) and social interaction (50) can improve cognitive ability. Therefore, increasing cognitive ability may enhance women’s subjective ability to purchase insurance.

**Figure 3:**
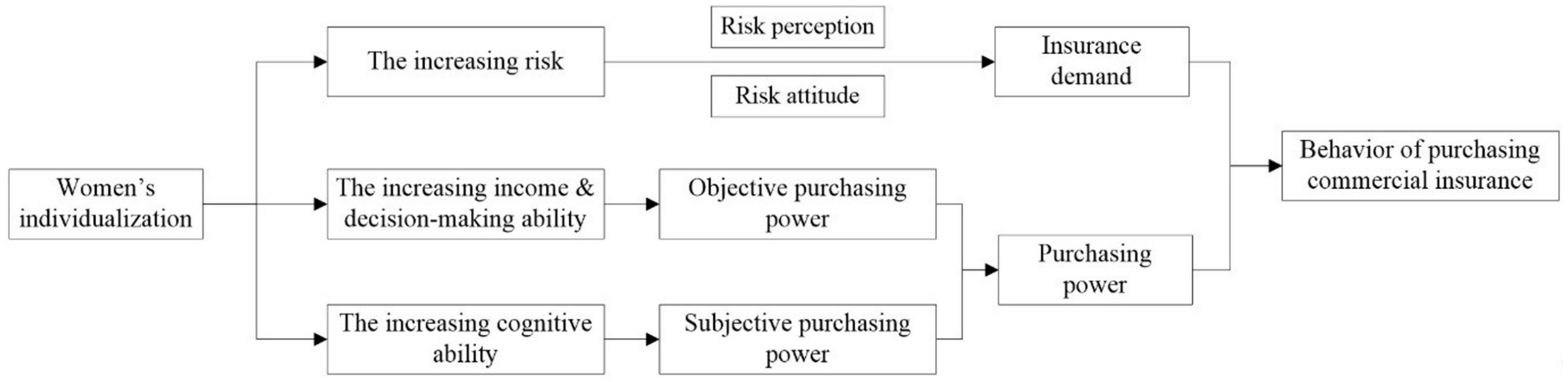
The transmission mechanism of women’s insurance purchasing behavior under individualization.

Figure 3 illustrates the transmission mechanism of women’s insurance purchasing behavior under individualization. Based on the above studies, we deem that women’s individualization would promote commercial insurance demands through three pathways: (1) increasing risk faced by women; (2) increasing income and decision-making power; and (3) increasing cognitive ability.

Based on the above theoretical analysis, we propose the following research hypotheses.

*Hypothesis 1*: Women’s individualization can significantly increase their probability of purchasing commercial insurance.

*Hypothesis 2a*: Individualization could encourage women to purchase insurance by improving women’s risk management awareness.

*Hypothesis 2b*: Individualization could encourage women to purchase insurance by improving women’s purchasing power.

*Hypothesis 2c*: Individualization could encourage women to purchase insurance by improving women’s cognitive ability.

As far as we know, there are only two studies relevant to our study. Zerriaa and Noubbigh (51) used data from 17 countries in the Middle East and North Africa to study the influencing factors of personal insurance demands and found that a high young dependency ratio inhibits the purchase of life insurance. Therefore, the individual independence of young people will increase their personal insurance needs. In addition, Chen (14) demonstrated that health insurance has a “marriage lock” function, which means that health insurance can reduce the risk of divorce for women, indicating that commercial insurance can serve as a means of individualized risk management tool for women. For the first time, we have empirically studied whether individualization encourages women to manage individualization risks by purchasing commercial insurance from the perspective of interdisciplinary fields such as psychology, sociology, and economics. To some extent, it compensates for the lack of relevant literature.

## 3 Data and method

### 3.1 Data and variables

This study used data from the CGSS, which was the earliest nationwide, comprehensive, and continuous academic investigation project in China and collected social, community, family, and individual data systematically and comprehensively. The China Survey and Data Center of the Renmin University of China has conducted this continuous cross-sectional survey of more than 10,000 households in various provinces and autonomous regions in mainland China every year since 2003. This study used the latest six-wave data (i.e., 2013, 2015, 2017, 2018, 2021, and 2023). After deleting the male samples, outliers, and missing values, we analyzed 26,442 samples. We used STATA 16.0 for data analysis.

This study focused on whether women’s individualization affects the purchase behavior of commercial insurance. To distinguish the influence of women’s individualization on different types of commercial insurance, we selected commercial medical and endowment insurance as dependent variables, respectively.

This study adopts gender equality consciousness as a proxy indicator for women’s individualization. Individualization includes multidimensional processes: individuals’ detachment from traditional structures, autonomous identity formation, and assertion of personal rights (1). In an individualized society, women have embraced new beliefs and norms, possess a strong awareness of equal rights, and do not agree with the old norm of “male superiority and female inferiority” (52). Crucially, the erosion of gender destiny beliefs (2), where women reject biologically fixed roles, signals the core of individualization. Meanwhile, gender equality consciousness can facilitate decision-making autonomy and social participation and can serve to measure the degree of female individualization to some extent. Therefore, aligning with You and Hao’s (52) practice, we operationalize women’s individualization through gender equality consciousness. Furthermore, to ensure the robustness of the empirical results, we also used another comprehensive indicator to measure “individualization” in the robustness test section.

Based on the existing literature, we set several control variables. First, we controlled the province, age, residence, and marital status as demographic factors that can have an impact on commercial insurance purchase behavior (53). Second, we controlled for income because it is a key factor affecting insurance purchasing behavior. Third, we controlled for education because it can affect financial literacy (53), risk attitude (10), and women’s independence consciousness (1, 52), which may influence commercial insurance purchases. Fourth, we controlled for health status, including physical and mental health, social insurance participation, including social medical insurance, and social endowment insurance that would affect the purchase of commercial insurance. When the independent variable was commercial medical insurance, we used social medical insurance as a control variable, and when the independent variable was commercial endowment insurance, we used social endowment insurance as a control variable.

Appendix Table A presents the definitions of dependent, independent, and control variables used in this study.

**Figure 4:**
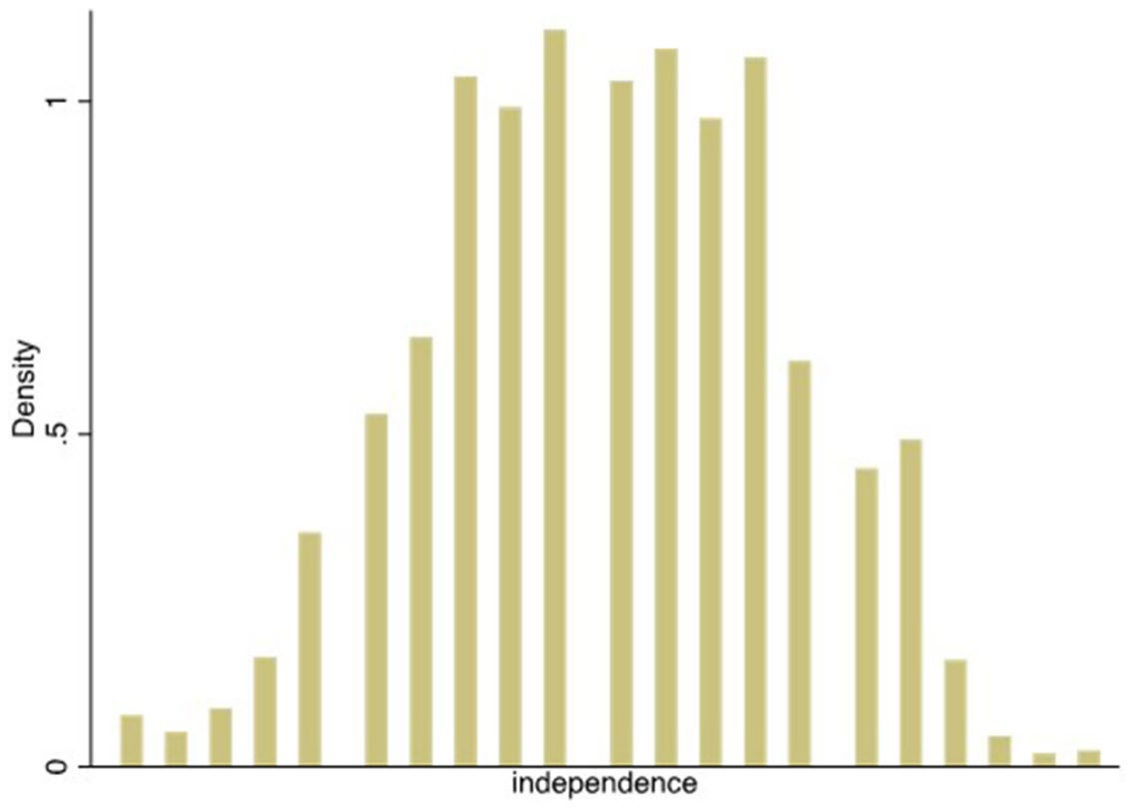
Probability density of independent variable.

Table 1 reveals the descriptive statistics for all samples, high individualization samples (independent variable is greater than or equal to 4), and low individualization samples (independent variable is less than 4). The average value of commercial medical insurance from high individualization samples was 0.151, which was significantly larger than the value of 0.074 for low individualization samples. The average value of commercial endowment insurance for high individualization samples was 0.091, which was significantly larger than the value of 0.053 for low individualization samples. The comparison results preliminarily confirmed the promotion effect of women’s individualization on commercial medical and endowment insurance. In addition, women with higher levels of individualization generally have higher levels of education, higher incomes, improved health conditions, and are younger. Figure 4 shows the probability density distribution of the independent variable, which tends to be a normal distribution.

**Table 1:** Description statistics.

Variable	All samples (*N* = 26,442)	High individualization samples (*N* = 11,830)	Low individualization samples (*N* = 14,612)	Difference
	Mean	Mean	Mean	Mean
Commercial medical insurance	0.109	0.151	0.074	0.077***
Commercial endowment insurance	0.070	0.091	0.053	0.038***
Province	14.577	14.08	14.979	−0.899***
Age	50.626	46.457	54.001	−7.544***
Age^2^	2,834.113	2,450.482	3,144.703	−694.222**
Education	0.174	0.291	0.079	0.212***
Income	7.602	8.186	7.130	1.056***
Physical health	3.493	3.662	3.356	0.306***
Mental health	3.808	3.905	3.729	0.176***
Residence	1.714	1.899	1.565	0.334***
Marital status	3.405	3.187	3.581	−0.395***
Social medical insurance	0.922	0.925	0.919	−0.005
Social endowment insurance	0.720	0.713	0.725	−0.013**

***p < 0.01, **p < 0.05. Both the linear and quadratic terms of the variable (age and age squared) were included in the regression model to capture potential non-linear relationships.

### 3.2 Empirical strategy

This section validates Hypothesis 1, proposed in the Literature Review and Theoretical Analysis section, through empirical models.

#### 3.2.1 Basic regression

As the dependent variables were binary, we chose the probit model as the basic model. The model settings are as follows:

(1) $$\mathrm{Pr}\left(\mathrm{Medical}{\mathrm{insurance}}_{i}=1\left|X_{i}\right.\right)=\Phi \left(\begin{array}{l} \alpha_{1}+\alpha_{2}{\mathrm{Individualization}}_{i}\\ +\sum_{j=1}^{11}\gamma_{j}{\mathrm{Control}}_{\mathrm{ij}}+\varepsilon_{i} \end{array}\right)$$

(2) $$\mathrm{Pr}\left(\mathrm{Endowment}{\mathrm{insurance}}_{i}=1\left|X_{i}\right.\right)=\Phi \left(\begin{array}{l} \beta_{1}+\beta_{2}{\mathrm{Individualization}}_{i}\\ +\sum_{j=1}^{11}\delta_{j}{\mathrm{Control}}_{\mathrm{ij}}+\epsilon_{i} \end{array}\right)$$

In the Equation 1, the binary variable $\mathrm{Medical}{\mathrm{insurance}}_{i}$ denotes whether individual i purchased commercial medical insurance. In the Equation 2, the binary variable $\mathrm{Endowment}{\mathrm{insurance}}_{i}$ denotes whether individual i purchased commercial medical insurance. $X_{i}$ represents the observable variables affecting individual i’s purchasing behavior of commercial insurance, including ${\mathrm{Individualization}}_{i}$ and ${\mathrm{Control}}_{\mathrm{ij}}$ . ${\mathrm{Individualization}}_{i}$ indicates the women’s individualization status. ${\mathrm{Control}}_{\mathrm{ij}}$ indicates the control variables. Both $\varepsilon_{i}$ and $\epsilon_{i}$ are residual errors.

#### 3.2.2 Endogenous analysis

To overcome potential endogeneity problems such as measurement error, omitted variables, and simultaneous causality in the basic regression, we applied instrumental variable (IV) and propensity score matching (PSM) regressions.

##### 3.2.2.1 PSM

To mitigate sample selection bias stemming from non-random distribution of women’s individualization traits, we employed PSM regressions. This approach approximates random assignment by pairing similar treatment group and control group based on their observable characteristics. We designated women exhibiting high individualization, defined as original autonomy scores of 4 or 5, as the treatment group; while women exhibiting low individualization with the scores of 1 and 3, as the control group. Propensity scores were derived from logistic regression incorporating all baseline covariates. In addition, we systematically implemented three matching methods, nearest neighbor, radius, and kernel matching, to enhance methodological rigor. Validation of covariate balance, common support, and treatment effects is reported in Section 4.2.1.

##### 3.2.2.2 IV

However, if the unobserved time-variant factors were correlated with both the degree of female individualization and the purchase of insurance, our estimate of the coefficient β_1_ would still be biased. To address endogeneity from unobserved confounders, we implemented an IV approach. This method isolates exogenous variation in women’s individualization by leveraging province-level averages of individualization as an instrument. The instrument satisfies the correlation requirements between the instrumental and endogenous variables because provincial cultural and economic conditions strongly correlate with individual autonomy levels. Crucially, it meets the requirement of exogeneity as aggregate provincial trends influence individual insurance decisions only through personal individualization pathways—province-wide averages cannot directly determine an individual’s insurance purchases. Statistical tests for instrument validity and explanatory variables endogeneity detection are detailed in Section 4.2.2.

## 4 Results

### 4.1 Basic results

We examined the impact of women’s individualization on purchases of commercial medical and endowment insurance. Table 2 presents the basic regression results. Column (1) shows that women’s individualization has a significantly positive impact on commercial medical insurance purchases. When the control variables were added in column (2), the coefficient of the independent variables was 0.095 and remained significant. After calculation, the corresponding marginal effect was 0.0143, thus indicating that women’s individualization can significantly increase the probability of purchasing commercial medical insurance by 1.143%. Columns (3) and (4) show that women’s individualization has a positive and significant impact on commercial endowment insurance purchases. When the control variables were added in column (4), the coefficient of independent variables was 0.068, and after calculation, the corresponding marginal effect was 0.00743, indicating that women’s individualization can significantly increase the probability of purchasing commercial endowment insurance by 0.743%. According to these regression results, women’s individualization can significantly encourage them to purchase commercial insurance, which is consistent with the conclusion drawn from the descriptive statistics. Hypothesis 1 is verified.

**Table 2:** Effect of women’s individualization on commercial insurance.

Variable	(1)	(2)	(3)	(4)
	Medical insurance	Medical insurance	Endowment insurance	Endowment insurance
Individualization	0.318***	0.095***	0.226***	0.068***
	(0.015)	(0.017)	(0.017)	(0.020)
Province		−0.010***		−0.010***
		(0.001)		(0.001)
Age		0.041***		0.053***
		(0.005)		(0.006)
Age^2^		−0.001***		−0.001***
		(0.000)		(0.000)
Education		0.584***		0.454***
		(0.030)		(0.034)
Income		0.022***		0.026***
		(0.003)		(0.004)
Physical health		0.058***		0.106***
		(0.013)		(0.014)
Mental health		0.029**		0.032**
		(0.013)		(0.014)
Residence		0.130***		0.110***
		(0.010)		(0.011)
Marital status		0.003		0.006
		(0.009)		(0.010)
Year		0.041***		0.016***
		(0.004)		(0.004)
Social medical insurance		−0.181***		–
		(0.041)		–
Social endowment insurance				0.123***
				(0.033)
R square	0.026	0.131	0.014	0.097
Observation	26,442	26,442	26,442	26,442

Robust standard errors in parentheses.

***p < 0.01, **p < 0.05. Both the linear and quadratic terms of the variable (age and age squared) were included in the regression model to capture potential non-linear relationships.

### 4.2 Endogeneity results

#### 4.2.1 PSM validation and treatment effect

Following nearest-neighbor, radius, and kernel matching implementations, all covariates achieved balanced distributions with standardized mean differences below 10%, thus indicating that the matching results met the balance requirement well and that the fitting degree of the treatment and control groups was better. While common support retention exceeded 99% of the original sample.

The result is shown in Table 3. For medical insurance coverage, the average treatment effect on treated individuals ranges between 0.034 and 0.036 standardized units across matching methodologies, with corresponding *t*-statistics ranging from 3.31 to 4.61 indicating statistical significance at the 1% level. Similarly, endowment insurance uptake demonstrates positive treatment effects between 0.019 and 0.027 standardized units, supported by *t*-values spanning 3.09 to 3.27 that likewise achieve conventional significance thresholds. These consistent findings across nearest-neighbor, radius, and kernel matching techniques confirm our basic results in Section 4.1.

**Table 3:** Effect of women’s individualization on commercial insurance-PSM regression.

Dependent variable	Nearest neighbor matching		Radius matching		Kernel matching	
	ATT	*t*-value	ATT	*t*-value	ATT	*t*-value
Medical insurance	0.036	3.31	0.036	4.61	0.034	4.29
Endowment insurance	0.027	3.10	0.021	3.27	0.019	3.09

#### 4.2.2 IV validation and estimation

Table 4 presents the regression results using the instrumental variable method. Diagnostic tests confirm the instrument’s strength: the first-stage F-statistic was 447.82 and 446.79 for the dependent variables of commercial medical and endowment insurance, respectively, rejecting weak instrument concerns. The Durbin–Wu–Hausman test statistic of 82.46 (*p* = 0.00), validating our endogeneity concerns. Most critically, the results of the two-stage regression showed that the coefficients of the independent variables were statistically significantly positive, thereby indicating that women’s individualization can significantly promote their purchase of commercial insurance.

**Table 4:** Effect of women’s individualization on commercial insurance-IV regression.

Variable	Independence	Medical insurance	Independence	Endowment insurance
	First stage	Second stage	First stage	Second stage
Individualization—IV	0.524***		0.518***	
	(0.032)		(0.032)	
Independence		1.540***		1.637***
		(0.183)		(0.207)
Control variable	Yes	Yes	Yes	Yes
F-test	447.82	—	446.79	—
Observation	26,442	26,442	26,442	26,442

Robust standard errors in parentheses.

***p < 0.01.

### 4.3 Robustness analysis

#### 4.3.1 Placebo test

**Figure 5:**
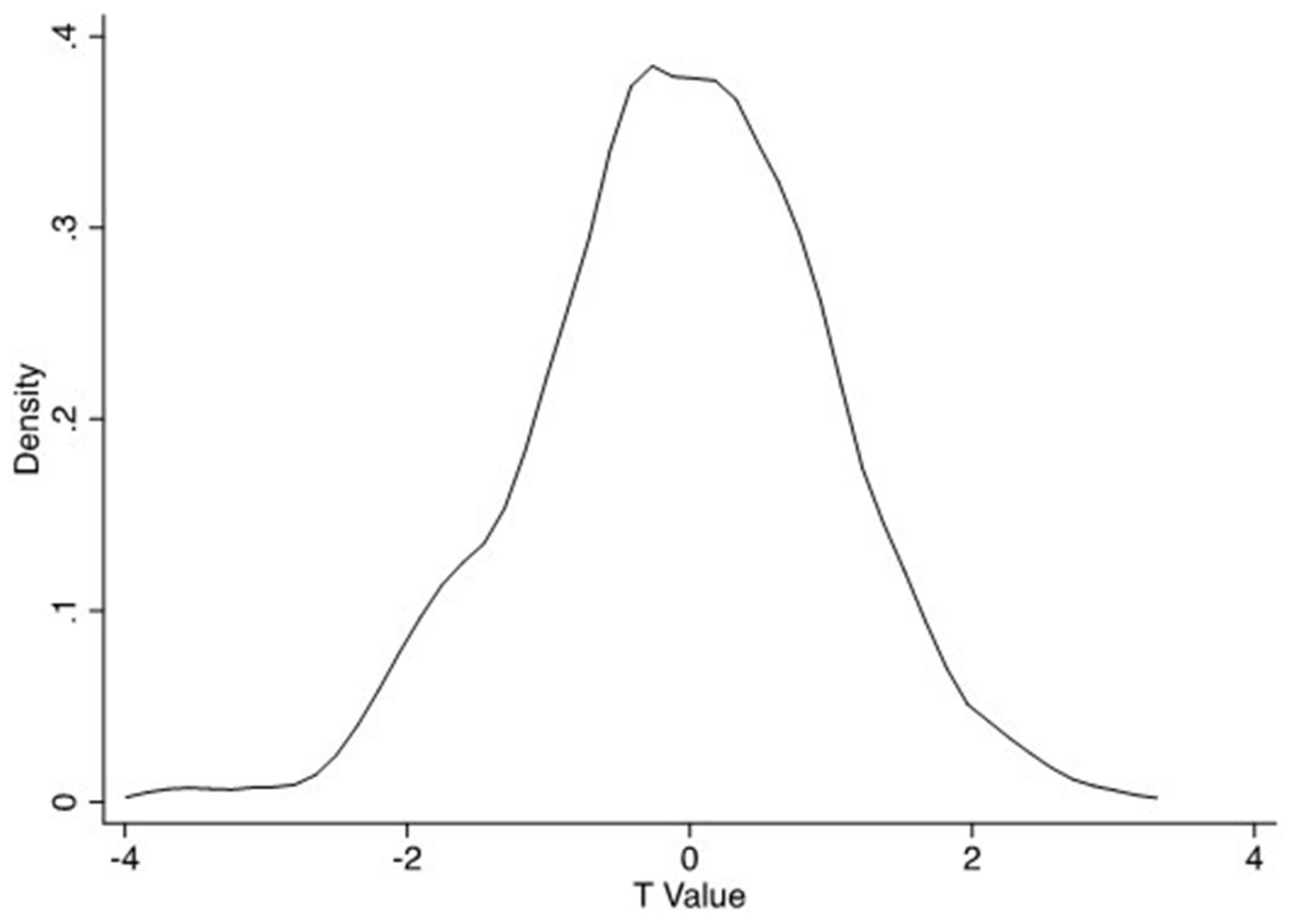
Placebo test (a).

In this sector, we adjusted variable individualization to binary variables, as in the above PSM regression. When the dependent variable was commercial medical insurance, the placebo test process was as follows: the number of valid samples was 26,442, including 11,830 samples of individualization and 14,612 samples of non-individualization. We randomly selected 6,537 samples from the whole sample and regarded them as samples of individualization; we also assigned the corresponding independent variable of individualization a value of 1. We regarded the remaining samples as samples of non-individualization and assigned the corresponding independent variable of individualization a value of 0. Thereafter, probit regression was performed according to the basic regression model, and the *t*-values corresponding to the regression coefficients of the independent variable of individualization were saved. We repeated this operation 500 times and then drew the saved probability density distribution of the *t*-values. Figure 5 illustrates that in most regression results, the *t*-value of the individualization coefficient was smaller than that of the individualization coefficient in the basic regression results (5.453), and most of the individualization coefficients were at an insignificant level.

**Figure 6:**
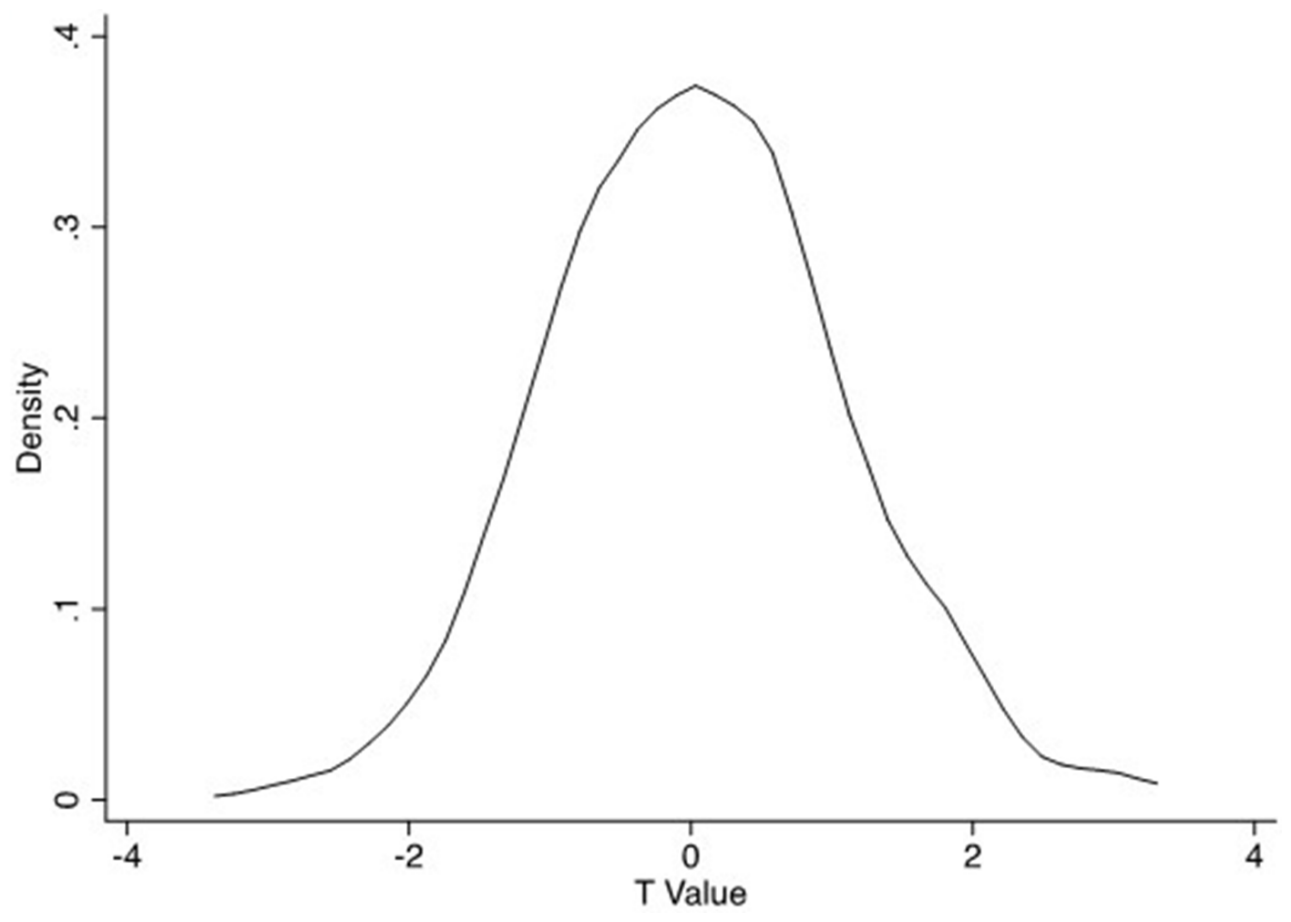
Placebo test (b).

When the dependent variable was commercial endowment insurance, the placebo test was similar to the process discussed above. Figure 6 illustrates that the *t*-value of the individualization coefficient in most of the regression results was smaller than that of the individualization coefficient in the basic regression results (3.489), and most of the individualization coefficients were at an insignificant level.

Overall, the placebo test results indicated that individualization can significantly encourage women to purchase commercial medical and endowment insurance.

#### 4.3.2 Changing estimation method

In this section, we employed three alternative estimation methods to conduct robustness checks. First, as the dependent variable was binary, the logistic model was used for estimation. Columns (1) and (2) in Table 5 suggests that the coefficients of the independent variable were significantly positive at 1%. Second, ordinary least squares (OLS) was also applied to estimate the basic model, yielding similar results, shown in Columns (3) and (4). Third, given that the CGSS constitutes non-longitudinal data precluding the use of individual fixed-effects models, we implemented robustness checks by controlling for time and province fixed effects. The results remain robustly consistent with our benchmark regression findings, as presented in Columns (5) and (6).

**Table 5:** Effect of women’s individualization on commercial insurance upon changing the estimation method.

Variable	Logistic		OLS		Fixed-effects	
	Medical insurance	Endowment insurance	Medical insurance	Endowment insurance	Medical insurance	Endowment insurance
Independence	0.187***	0.141***	0.014***	0.007***	0.061***	0.037*
	(0.034)	(0.040)	(0.003)	(0.002)	(0.018)	(0.020)
Control variables	Yes	Yes	Yes	Yes	Yes	Yes
R square	0.131	0.098	0.296	0.053	0.147	0.120
Provincial FE	No	No	No	No	Yes	Yes
Time FE	No	No	No	No	Yes	Yes
Observation	26,442	26,442	26,442	26,442	26,159	26,159

Robust standard errors in parentheses.

***p < 0.01, *p < 0.1.

#### 4.3.3 Adjusting samples

To avoid the influence of extreme values on the regression results, we minorized the data. Table 6 reveals that after cutting the first and last 2.5% of the data, the coefficients of the independent variables are still significantly positive at 1%. In addition, considering that insurance companies may reject older adults after reaching 65 due to their health status, we deleted the samples of those over 65. Table 6 indicates that the coefficients of independent variables were still significant at the 1% level.

**Table 6:** Effect of women’s individualization on commercial insurance after adjusting samples.

Variable	Medical insurance	Endowment insurance	Medical insurance	Endowment insurance
	Winsorize		Minus samples of those over 65 years	
Independence	0.110***	0.071***	0.107***	0.080***
	(0.020)	(0.022)	(0.019)	(0.021)
Control variables	Yes	Yes	Yes	Yes
R square	0.132	0.097	0.118	0.097
Observation	24,918	24,918	21,019	21,019

Robust standard errors in parentheses.

***p < 0.01.

#### 4.3.4 Replacing the independent variable

Individualization in the basic model was replaced with Individualization-rep. The changed variable Individualization-rep is the mean value of the answers to five questions about gender identity in the CGSS2017 data. The definition of the variable individualization-rep is provided in Appendix Table B.

Table 7 suggests that after replacing the independent variable, the coefficients of the independent variables in the regression results were significantly positive. The results were consistent with those of the basic regression.

**Table 7:** Effect of women’s individualization on commercial insurance upon replacing independent variables.

Variable	Medical insurance	Medical insurance	Endowment insurance	Endowment insurance
Individualization-rep	0.259***	0.097**	0.206***	0.090**
	(0.036)	(0.041)	(0.036)	(0.041)
Control variables	No	Yes	No	Yes
R square	0.053	0.187	0.035	0.128
Observation	1,724	1,724	1,724	1,724

Robust standard errors in parentheses.

***p < 0.01, **p < 0.05.

### 4.4 Mechanism analysis

This section verifies the mechanism based on the research Hypothesis 2 proposed in the Literature Review and Theoretical Analysis section.

Referring to Qu and Luo (54), the bootstrap method based on bias correction was used to investigate the mediation effect. We carried out 500 repeated samplings with returns to obtain bootstrap samples similar to the original samples. Table 8 presents the results of the mediating effects for risk management awareness, purchasing power, and cognition ability.

**Table 8:** Mediating effect analysis.

Mediating variable	Insurance	Effect	Coefficient	Standard error	95% confidence interval	
					Lower limit	Upper limit
Risk management awareness	Medical insurance	Indirect	0.00206***	0.00026	0.00160	0.00263
		Direct	0.01198***	0.00279	0.00648	0.01738
	Endowment insurance	Indirect	0.00119***	0.00021	0.00078	0.00162
		Direct	0.00637***	0.00237	0.00202	0.01094
Purchasing power	Medical insurance	Indirect	0.00158***	0.00026	0.00113	0.00211
		Direct	0.01428***	0.00282	0.00846	0.01941
	Endowment insurance	Indirect	0.00136***	0.00021	0.00095	0.00179
		Direct	0.00728***	0.00250	0.00239	0.01264
General cognitive ability	Medical insurance	Indirect	0.00278***	0.00029	0.00223	0.00344
		Direct	0.01109***	0.00269	0.00569	0.01641
	Endowment insurance	Indirect	0.00169***	0.00023	0.00122	0.00216
		Direct	0.00634***	0.00238	0.00149	0.01064
Insurance knowledge cognition	Medical insurance	Indirect	0.00248***	0.00043	0.00155	0.00330
		Direct	0.01069***	0.00277	0.00517	0.01590
	Endowment insurance	Indirect	0.00171***	0.00313	0.00115	0.00234
		Direct	0.00563***	0.00252	0.00104	0.01094

***p < 0.01.

First, the frequency of exercise was used to measure women’s awareness of risk management. As Table 8 reveals, the coefficients of the indirect effects were all significantly positive, and 0 was not included in the 95% confidence interval. The results indicate that women’s individualization may affect commercial insurance purchasing behavior through risk management awareness. Hypothesis 2a is verified.

Second, we used income as an alternative variable of purchasing power to examine whether purchasing power is a mediating variable. In Table 8, the coefficients of the indirect effects were all significantly positive, and 0 was not included in the 95% confidence interval. Hence, we can conclude that women’s individualization may promote their purchase of commercial insurance by improving their purchasing power. Hypothesis 1 is verified.

Finally, we examined the mediating effects of cognitive ability across dual dimensions: (1) general cognitive ability, proxied by Mandarin proficiency scores (2–10 discrete-continuous scale); (2) insurance knowledge cognition, operationalized through financial literacy (financial investment status) (55). We then separately tested the mediation effects by incorporating each proxy variable into the analytical model. As Table 8 indicates, both general cognitive ability and insurance knowledge cognition exhibit statistically significant mediation effects. This indicates that female individualization influences insurance purchase decisions through a dual cognitive pathway mechanism. Hypothesis 2c is verified.

The definitions of the mediating variables used in this part (except for the mediating variable income) are provided in Appendix Table B.

### 4.5 Heterogeneity analysis

This study used the grouping regression method to analyze the heterogeneity, in three dimensions, including region, job status, wealth level, physical health, and age. Among them, the regional division standard comes from the National Bureau of Statistics of China. Table 9 presents the results: (1) the coefficients of the eastern and central regions are significantly positive, thus indicating that women’s individualization could significantly promote the purchase of commercial insurance in the eastern and central regions. (2) Individualization can encourage working women to purchase commercial insurance significantly. (3) We measured women’s wealth based on whether they owned real estate and conducted grouping regression. The results show that individualization can encourage women with higher wealth levels to purchase commercial medical and endowment insurance more significantly. The findings reveal an important nuance: while individualization similarly enhances risk awareness and financial understanding across wealth groups, financial constraints prevent low-wealth women from translating these benefits into actual insurance purchases. (4) Physical health is constructed as the categorical variable, with values ranging from 1 to 5, indicating very unhealthy to very healthy, respectively. If the physical health value is greater than 3, it is defined as a high health level, while other values are defined as a low health level. The results suggest that individualization will encourage women to purchase commercial medical insurance, regardless of their health status. However, individualization can only encourage women to purchase pension insurance when their health level is low. (5) According to the World Health Organization’s 2021 age classification standards, individuals under 44 are considered young, those between 45 and 59 are considered middle-aged, and those over 60 are considered older adults. The coefficients are significantly positive for young and older adults.

**Table 9:** Heterogeneity analysis.

Grouping variable		Control variable	Medical insurance	Endowment insurance
Region	Eastern region	Yes	0.096***	0.047*
			(0.024)	(0.026)
	Central region	Yes	0.092***	0.123***
			(0.033)	(0.038)
	Western region	Yes	0.062	0.131
			(0.040)	(0.047)
Job status	Having a job	Yes	0.079***	0.100***
			(0.024)	(0.027)
	Having no job	Yes	0.105***	0.039
			(0.026)	(0.029)
Wealth level	Having real estate	Yes	0.086***	0.062***
			(0.018)	(0.020)
	Having no real estate	Yes	0.236***	0.181*
			(0.065)	(0.074)
Physical health	High	Yes	0.091***	0.059**
			(0.022)	(0.025)
	Low	Yes	0.099***	0.087***
			(0.029)	(0.032)
Age	Youth	Yes	0.095***	0.056*
			(0.027)	(0.031)
	Middle age	Yes	0.116***	0.141***
			(0.029)	(0.033)
	Old age	Yes	0.066*	0.013
			(0.037)	(0.040)

Robust standard errors in parentheses.

***p < 0.01, **p < 0.05, *p < 0.1.

## 5 Discussion

The basic results indicate that women’s individualization can significantly increase the probability of purchasing commercial medical insurance by 1.430% and increase the probability of purchasing commercial endowment insurance by 0.743%. Although there are few studies directly on the impact of female individualization on insurance demand, our conclusion is consistent with some relevant literature. Zerriaa and Noubbigh (51) demonstrated that a high young dependency ratio inhibits the purchase of life insurance. Thus, the individual independence of young people will increase their personal insurance needs. In addition, health insurance has a “marriage lock” function, which means that it can reduce the risk of divorce for women (14), indicating that commercial insurance can serve as a tool for individualized risk management for women.

In the mechanism analysis, we confirmed the mediating effects of recognition risk management awareness, purchasing power, and cognition ability. This result is consistent with the findings of existing literature and our speculation. First, as we mentioned in the Introduction section, women’s individualization creates various risks for them, which could be harmful to their physical and mental health (20, 25). In addition, women are more sensitive to risk (9) and are found to be more risk-averse (10). Thus, risk management awareness can have a mediating effect on promoting women’s purchase of insurance. Second, with individualization, women generally participate in social work, and their income increases. Purchasing power is an important factor affecting insurance purchasing behavior. Therefore, the individualization of women can promote their purchase of commercial insurance by increasing their income. Thirdly, the result indicates that female individualization influences insurance purchase decisions through a dual cognitive pathway mechanism. Specifically, general cognitive ability represents foundational information processing capacity, which enhances the efficiency of acquiring insurance-related information; insurance knowledge cognition embodies insurance-specific knowledge in insurance investments, directly improving the quality of insurance decision-making. Both serve as valid mediators through which female individualization affects insurance decisions. As discussed in the Introduction section, we believe that individualized women tend to have a higher cognitive ability, which may encourage women to better understand the function of insurance and manage risk through insurance.

In the heterogeneity analysis, we find that women’s individualization plays a greater role in promoting commercial insurance demands among those with jobs, high wealth levels, and worse health status, young and middle-aged, and in China’s central and eastern regions. (1) According to the regression results, individualization can encourage working women to purchase commercial insurance more significantly. It can be explained by the fact that women who participate in social work usually face more work pressure and work–family conflict (7, 26), which may result in many risks (e.g., health risks). In addition, employed women tend to have more advanced knowledge levels and more opportunities to contact the outside world; thus, they have a better understanding of insurance, an improved ability to make family decisions (42), and greater purchasing power. (2) Individualization can more significantly encourage women at higher wealth levels to purchase commercial insurance. This result is consistent with Lee’s (56) findings that wealth levels can drive insurance purchases. For those facing financial limitations, we observe that improved insurance awareness and enhanced cognitive ability does not translate into significantly higher purchase rates. This disconnect underscores the importance of developing tailored policy solutions that help economically vulnerable yet highly individualized women overcome barriers to insurance access. (3) Individualization can more significantly encourage women who have a worse health status. A possible explanation is that women with poor physical conditions may expect an increase in aging and health expenses and require pension insurance as an income guarantee. (4) Individualization will encourage young and middle-aged women to purchase commercial health and pension insurance, but it has no impact on insurance purchases by the older adults. Possible explanations are the low insurance cognition among the older adults and the high premium rates. (5) In China, the eastern region has the highest level of economic and social development, followed by the central region, and the western region has the lowest level of development. The regression shows that individualization can more significantly encourage women in China’s central and eastern regions. It may be because deep traditional beliefs in the Western region restrain the demand for insurance (57). The central and eastern regions have a higher level of economic development, social development, and women’s individualization.

## 6 Conclusion

Women’s individualization, which enables them to become unshackled from their family bondage and become more independent, produces a variety of personal risks. This study explores whether female individualization has promoted women to manage risks through commercial insurance as a risk management tool. Based on CGSS 2013, 2015, 2017, 2018, 2021and 2023 data, this study analyzed the influence of women’s individualization on commercial insurance purchases using the probit model, instrumental variable method, propensity score matching, and other methods. The results indicated that women’s individualization can significantly increase the probability of purchasing commercial medical insurance by 1.430% and can increase the probability of purchasing commercial endowment insurance by 0.743%%. Individualization was found to encourage women to purchase insurance by improving their risk management awareness, purchasing power, and cognitive ability. Heterogeneity analysis showed that women’s individualization played a greater role in promoting commercial endowment and medical insurance among those women with jobs, higher wealth levels, worse health status, young and middle-aged, and in China’s central and eastern regions.

To the best of our knowledge, this research is the first to empirically study whether individualization encourages women to purchase commercial insurance. We also explored how individualization promotes women purchasing commercial insurance through theoretical analysis and moderating effects. We expanded the existing social individualization theory and promoted the integration of individualization and risk management theories.

This study found that individualization creates many risks for women and increases their insurance demand. The findings have several practical implications. (1) Policymakers are advised to prioritize insurance subsidies for vulnerable women and adapt regulations to regional market needs. For example, subsidize micro-insurance products targeting low wealth women workers, particularly those showing high individualization traits. (2) Insurers should innovate tailored products addressing individualized women’s distinct risk profiles. Specifically, for employed women, cooperate with enterprises to offer group insurance preferences and promote work–life risk-related products in workplaces; for women with higher wealth, develop diverse high-end commercial medical insurance and endowment insurance; for women with poorer health, develop more affordable health insurance with relaxed underwriting and set up consulting channels to assist them; for younger and middle-aged women, popularize insurance knowledge online and in communities, focusing on products covering career and family risks; for women in central and eastern China, expand service coverage, optimize offline outlets, and improve purchase and claim convenience. (3) Women should proactively mitigate personal risks through tailored insurance solutions, such as transferring health risks via commercial medical insurance, hedging against retirement income risks by converting wealth into annuities. (4) Researchers are offered a novel framework linking social transformation to financial behavior, urging further cross-cultural investigation.

This study confirms that women’s individualization significantly increases commercial insurance demand in China through enhanced risk awareness, purchasing power, and cognitive ability. However, we should mention that these findings operate under the assumption: functioning insurance markets and accessible insurance product exist in sampled regions.

However, this study has some other limitations. First, according to the literature, the decision-making power of women may promote family consumption, but we were unable to verify it as an intermediary mechanism due to the limitations of the data used. Second, the CGSS has relevant indicators regarding whether the respondents purchased commercial medical or endowment insurance only, which results in our inability to study the influence of women’s individualization on the purchase of other types of commercial insurance. Third, general cognition ability encompasses mathematical skills, verbal fluency, and recall skills (46), among others. However, as we are limited by the available data, we were only able to include indicators of verbal skills to measure general cognition ability.

In future research, we can further investigate the impact of female individualization on commercial insurance purchases from the aspect of mechanism analysis and specific insurance types. In addition, female individualization is only one perspective of social individualization, and how to deal with other individualization risks is also worth studying.

## Data Availability

The datasets for this study can be found in the Chinese General Social Survey: http://cgss.ruc.edu.cn.

## Footnotes

^1^Data from the fourth Chinese Women’s Social Status Survey, Fourth Chinese Women’s Social Status Survey leading group office, 2020.

^2^Data from Statistical Monitoring report on the final phase of program for the Development of Chinese Women (2011–2020), National Bureau of Statistics (China).

^3^Evidenced by women’s 154 daily housework minutes, doubling male burdens. Data from the fourth Chinese Women’s Social Status Survey, the fourth Chinese Women’s Social Status Survey leading group office, 2020.

^4^Data from the White Paper on Women’s Health and Risk Management in China, Fudan Development Institute and Manulife-Sinochem Life Insurance Co. Ltd., 2019.

^5^Data is from the 2020 Statistical Bulletin on The Development of Civil Affairs, Ministry of Civil Affairs of the People’s Republic of China, 2020. Divorce rate = number of divorces in the current year/total population in the current year * 1000%.
