# Supplementary material for: The impact of women’s individualization on commercial insurance purchase behavior: evidence from China
Source: Front Public Health. 2025 Oct 7;13:1627096. doi: 10.3389/fpubh.2025.1627096 (PMC12537675; doi:10.3389/fpubh.2025.1627096)
Supplement: Supplementary file 1 [file Table_1.docx]

Supplementary Material

# Appendix Table A. Variable definition- dependent, independent, and control variables

| Variable | Variable definition |
| --- | --- |
| Dependent variables | |
| Commercial medical insurance | This variable is equal to 1 if the respondent has purchased commercial medical insurance and 0 otherwise. |
| Commercial endowment insurance | This variable is equal to 1 if the respondent has purchased commercial endowment insurance and 0 otherwise. |
| Independent variable | |
| Individualization of women | Respondents were asked five questions. The independent variable was the average value of the five questions.  Do you agree that men should put their career first and women their family first?  Do you agree or disagree that men are naturally more capable than women?  Do you agree it is better to marry well than to do well?  Do you agree that women should be fired first in a bad economy?  Do you agree that husband and wife should share housework equally?  The scores of the five questions are: Completely agree =1, relatively agree =2, indifferent/neither agree nor disagree =3, relatively disagree =4, completely disagree =5. |
| Control variables | |
| Province | Assignment 1–31. |
| Year | Year of the survey. |
| Age | Respondent’s age. |
| Age^2^ | Respondent’s age square. |
| Education level | College or above =1, otherwise =0. |
| Income | Take the logarithm of the respondent’s total income last year after adding 1. |
| Physical health | How do you feel about your current physical health?  Very unhealthy = 1, unhealthy = 2, general health status = 3, healthy = 4, very healthy = 5. |
| Mental health | In the past 4 weeks, how often did you feel depressed or depressed?  Always =1, often =2, sometimes =3, rarely =4, never =5 |
| Residence | Agricultural household registration=1, non-agricultural household registration=2, blue print household registration=3, resident household registration (formerly agricultural household registration)=4, resident household registration (formerly non-agricultural household registration)=5. |
| Marital status | Unmarried=1, cohabiting=2, first marriage with spouse=3, remarriage with spouse=4, separation without divorce=5; divorce=6, widowhood=7. |
| Social medical insurance | This variable is equal to 1 if the respondent was covered by a social medical insurance scheme and 0 otherwise. |
| Social endowment insurance | This variable is equal to 1 if the respondent was covered by a social medical insurance scheme and 0 otherwise. |

# Appendix Table B. Variable definition- mediating variables , Individualization-rep

| Variable | Variable definition |
| --- | --- |
| Individualization-rep | Respondents were asked five questions. Variable individualization-rep's value is the average value of the five questions.  Do you agree that it is more important for a wife to help her husband’s career than to pursue her own?  Do you agree that the husband’s responsibility is to earn money and the wife’s responsibility is to take care of the family?  Do you agree that mothers should not go out to work until their children go to school?  Do you agree that men should do more housework than they do now?  Do you agree that in a bad economy, women should be fired first?  The scores of the five questions are: Completely agree =1, strongly agree =2, agree to some extent =3, doesn’t matter/ neither agree nor disagree =4, disagree to some extent =5, strongly disagree =6, completely disagree =7. |
| Risk management awareness | In the past year, do you often take part in physical exercise in your spare time? Every day or several times a week =1; Several times a month, several times a year or less, never =0 |
| General cognitive ability | Listening and speaking ability of Mandarin, assignment 2–10, the higher the value, the stronger the ability. |
| Insurance knowledge cognition | Do you have any investments such as stocks, funds, bonds, futures, warrants, or foreign exchange? Yes=1, No=0 |
